# The new reporting obligation for Respiratory Syncytial Virus (RSV) in Germany – a critical view

**DOI:** 10.3205/dgkh000475

**Published:** 2024-04-17

**Authors:** Ursel Heudorf, Anne Marcic, Katrin Simone Steul

**Affiliations:** 1 Institute of Hygiene and Environmental Medicine, Justus Liebig University Giessen, Germany; 2 Public Health Department of the State Capital Kiel, Germany; 3 Institute of Occupational, Social and Environmental Medicine, University Medical Center of the Johannes Gutenberg University Mainz, Germany

**Keywords:** respiratory syncytial virus (RSV), mandatory reporting, infection surveillance, RSV sentinels, public health authorities

## Abstract

**Background:**

In summer 2023, mandatory reporting of respiratory syncytial virus (RSV) by name was introduced in Germany. The stated objectives were:

1. to improve the database to prevent overburdening of the healthcare system,
2. to implement targeted, early investigation and action by local health authorities to prevent further spread, and
3. to assess vaccines after the expected approval of RSV vaccination.

**Methods:**

These objectives are examined against the background of data from mandatory reporting of RSV in the German federal state of Saxony, which has been required since 2002, and the data from the ARE (acute respiratory diseases) survey in Germany, considering

1. the basic legal requirements and options of the Infection Protection Act,
2. the requirements of the EU Commission for the collection of data on infectious diseases and the recommendations by experts of the European Centre for Disease Prevention and Control (ECDC),
3. the options for individual or general preventive measures by the health authorities and
4. previous experience with the evaluation options of the reported data (especially regarding the effectiveness of vaccinations).

**Results and discussion:**

An extrapolation of the previously reported data from Saxony to the whole of Germany shows that over 100,000 reports per year must be expected (more than the reports of both rota and noroviruses together). Neither the requirements of the EU Commission nor the views of an expert group of the ECDC recommend mandatory RSV reporting. Mandatory reporting by name is also not appropriate from a legal perspective. A sentinel, which is also better suited to assessing vaccinations, would be more appropriate to avoid unnecessarily overburdening the health authorities. In addition, initial experience with wastewater sentinels for RSV has shown that they may be used to record local and regional RSV infections – albeit without information on the severity of the disease and thus the burden on the healthcare system.

Against this background, mandatory reporting of RSV does not appear to be appropriate. Instead, the existing sentinels should be continued and further expanded, possibly supplemented by RSV wastewater monitoring.

## Zusammenfassung

**Hintergrund:**

Im Sommer 2023 wurde in Deutschland eine namentliche Meldepflicht für Respiratory Syncytial Virus (RSV) eingeführt. Als Ziele wurden angegeben:

1. Verbesserung der Datengrundlage (zur Verhütung der Überlastung des Gesundheitssystems),
2. Durchführung zielgerichteter und frühzeitiger Ermittlung und Maßnahmen der Gesundheitsämter vor Ort zur Verhinderung der Weiterverbreitung und
3. Beurteilung der Impfstoffe nach der zu erwartenden Zulassung einer RSV-Impfung.

**Methoden:**

Diese Ziele sollen vor dem Hintergrund der Daten der seit 2002 bestehenden RSV-Meldepflicht in Sachsen sowie der Daten aus dem Survey für Akute Respiratorische Erkrankungen (ARE-Survey) betrachtet werden. Berücksichtigt werden

1. rechtliche und fachliche Anforderungen und Möglichkeiten des Infektionsschutzgesetzes,
2. Anforderungen der EU-Kommission sowie der Expertengruppe der ECDC zur Erfassung von Infektionserkrankungen,
3. Möglichkeiten für Präventionsmaßnahmen durch die Gesundheitsämter und
4. die bisherigen Erfahrungen zu den Bewertungsmöglichkeiten der Meldedaten (insbesondere im Hinblick auf die Effektivität vom Impfungen)

**Ergebnisse und Diskussion:**

Eine Hochrechnung der bisherigen Meldedaten aus Sachsen auf ganz Deutschland zeigt, dass mit über 100.000 Meldungen pro Jahr gerechnet werden muss (mehr als die Summe der Meldungen für Rota- und Noroviren). Weder von der EU-Kommission noch gemäß der Auffassung der EU-Experten wird eine RSV-Meldepflicht empfohlen. Eine namentliche Meldepflicht ist auch aus rechtlicher Sicht nicht angezeigt. Hier wäre – um die Gesundheitsämter nicht unnötig zu belasten – ein Sentinel, das auch zur Beurteilung von Impfungen besser geeignet ist, sinnvoller. Darüber hinaus haben erste Erfahrungen mit Abwassersentinels bzgl. RSV gezeigt, dass hiermit lokale und regionale RSV-Infektionsgeschehen erfasst werden können – allerdings ohne Angaben zur Erkrankungsschwere und damit zur Belastung des Gesundheitssystems.

Vor diesem Hintergrund erscheint die Meldepflicht für RSV nicht sachgerecht. Stattdessen sollten die vorhandenen Sentinels weiter aufgestockt und fortgeführt werden, ggf. ergänzt durch RSV-Abwassermonitoring.

## Introduction

Respiratory syncytial virus (RSV) is the most common cause of lower respiratory tract infections in children, leading to annual epidemics worldwide [1]. Bronchiolitis caused by RSV in infants and young children often requires hospitalization and even intensive care [2]. Premature babies, newborns and young infants as well as children with chronic lung disease (e.g., interstitial lung disease, cystic fibrosis, congenital respiratory anomalies), congenital heart disease, neuromuscular diseases, severe immunodeficiencies, immunosuppressive therapy, or chromosomal aberrations such as trisomy 21 have a greater risk of a severe course [3]. 

The disease usually peaks in mid-winter (January/February). As a result of the SARS-CoV-2 pandemic and the associated contact restrictions, almost no RSV infections were observed in winter 2020/2021. In the following years, however, there were many more RSV cases than in the pre-pandemic years. There has been a shift towards occurrences in late fall to early winter (November/December) and illnesses also in older infants, but without a change in the severity of the disease [1], [4], [5]. The following causes were discussed: change in immunity of vulnerable groups due to reduced circulation of RS viruses during the pandemic, interactions between RSV and SARS-CoV-2, change in patient health seeking behavior during the pandemic [6].

In the summer of 2023, reporting obligation of RSV by name was introduced in Germany [7]. The justification was: RSV has led to a high number of sick children in the past two cold seasons and to a significant overload of pediatric clinics nationwide. As the most common respiratory pathogen in young children and due to progress in vaccine and prophylaxis development, RSV is becoming increasingly important in international health surveillance. In Germany, there is so far no obligation to report RSV in accordance with the IfSG – only in Saxony is there an obligation to report RSV when the pathogen is detected; this has been effective since 2002. The incidence of infections in 2022/2023 illustrated that the data basis needs to be improved to allow early detection of possible overloads in the healthcare system. The reporting obligation therefore not only serves to collect epidemiological data, but also to enable public health services to perform targeted, early investigations and take steps on site in order to contain an outbreak and prevent further spread. In addition, the prophylaxis of severe RSV infection for vulnerable groups could be improved by the timely administration of monoclonal antibodies. It is foreseeable that RSV vaccines in Germany will be approved. The additional information from the thus reported data would also be helpful for the assessment of the vaccines and the orientation of vaccination strategies. To enable comparisons with periods prior to the introduction of the vaccines, a rapid introduction of mandatory reporting makes sense [8].

The goals and objectives of introducing this new RSV reporting requirement, i.e., to

- improve the data basis (to prevent overburdening of the healthcare system),
- carry out targeted and early investigation and take measures to prevent further spread,
- evaluate vaccines following the expected approval of an RSV vaccine

are discussed against the background of the data from obligatory reporting of RSV in Saxony and the data from the ARE (acute respiratory diseases) surveys. The following are taken into considerating:

1. requirements and options of the Infection Protection Act,
2. requirements of the EU Commission for recording infectious diseases, as well as the recommendations from experts of the European Centre for Disease Prevention and Control (ECDC),
3. possibilities for individual or general preventive measures by the health authorities, and
4. experience gained to date regarding the possibilities for evaluating the data from mandatory reporting with particular regard to the effectiveness of vaccinations.

## Materials and methods

The mandatory reporting data from Saxony [9] were obtained from the homepage of the Robert Koch Institute Berlin using a Survstat query [5]. The data from the ARE surveys were taken from the surveillance data of the ECDC [10], to which the ARE surveillance data in Germany [11] are forwarded.

The evaluation was based on the requirements for obligatory reporting in the Infection Protection Act (IfSG and commentary Bales/Baumann), the relevant decisions of the EU Commission [12] [13], and recommendations of an expert group of the ECDC [14].

## Results

### Previous data on RSV from Germany

RSV has been notifiable in Saxony since 2002 [9]. The data as of 2013 are currently available on the Robert Koch Institute’s (RKI) SURVStat server, but are not described and discussed in the Infectious Disease Epidemiology Yearbook [15]. The State Research Institute of Saxony has published selected results of this reporting obligation in several reports [16]. In addition, RSV infections have been recorded as part of ARE surveillance since 2011 and published in the regular weekly reports [11].

In Saxony, after a few individual reports in 2015 and before, approximately 2,493 cases were reported in 2016, 2,194 of which were in under 5-year-olds (Table 1 (Tab. 1)). This corresponds to an incidence of 61/100,000 in the total population and 1,183/100,000 in under 5-year-olds. In the following years, there was a significant increase in reports, interrupted only by the first COVID-19 pandemic year 2020. By 2021/2022, reports had more than doubled to 6,000, corresponding to an incidence of approx. 150/100,000. Incidences of over 2,500/100,000 (2021) were obtained in the 0–4 age group (Table 1 (Tab. 1)). If the incidence of 150/100,000 from Saxony is extrapo-lated to the Federal Republic of Germany, more than 120,000 reports per year can be expected under the current conditions.

Although over 70% of the reports concerned infants and young children, severe courses of the disease as well as deaths were documented among adults, particularly in the elderly group. In 2019, 23 people aged 50–92 died in Saxony (median 83.5 years); in 2021 11 deaths occurred in this age group (median 86 years) [16]. Infants and young children are therefore mainly affected by the infection, but senior citizens are at particular risk.

**Figure 1:**
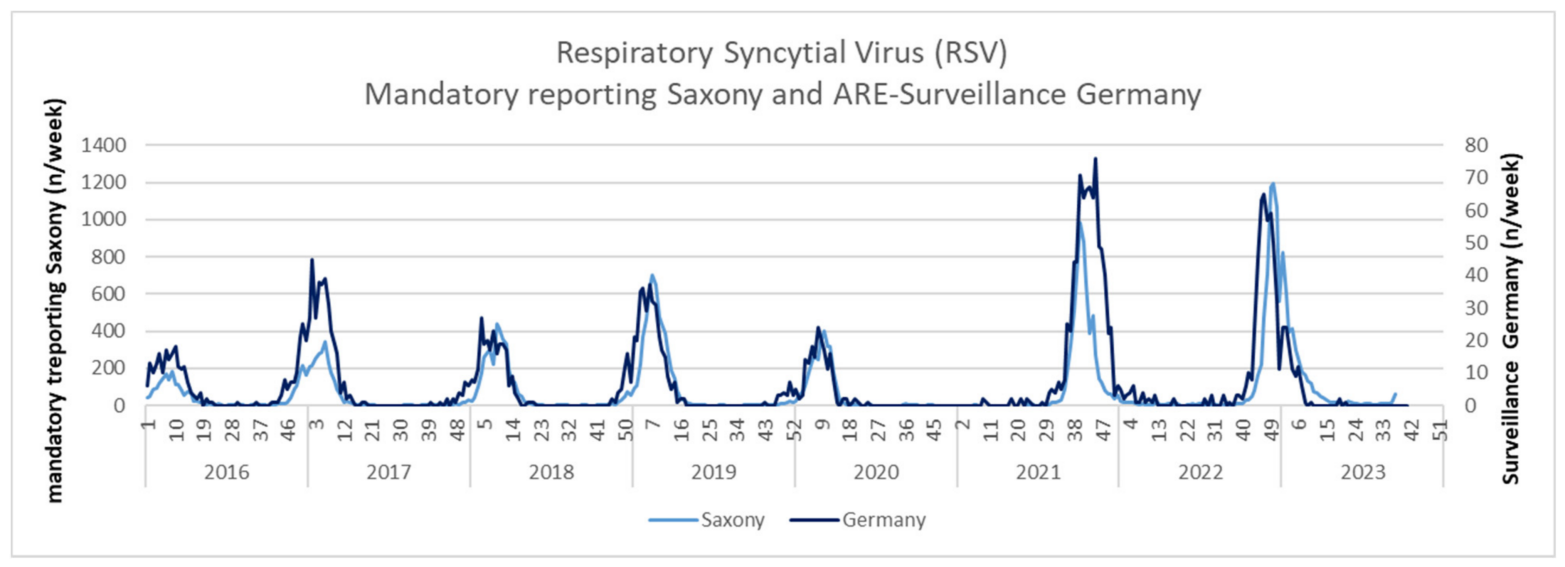
Mandatory reporting data for RSV detections in Saxony per calendar week (light blue curve) compared with the data collected as part of ARE surveillance (dark blue curve)

The analysis of reports from Saxony by reporting week shows a shift towards earlier months since 2021. Until the start of the pandemic in March 2020, most RSV reports occurred in the winter months of January to March, with a peak in February. In 2021, many infections then occurred for the first time in the fall and in 2022, particularly in December, with significantly higher incidences than in the previous years. The reporting data per calendar week from Saxony are shown in Figure 1 (Fig. 1). 

Figure 1 (Fig. 1) also encompasses the ARE surveillance data. The congruence between the two curves is remarkable, although the data from ARE surveillance in Germany only accounts for less than 10% of the reported numbers from Saxony. Thus, the earlier start of the RSV season in autumn 2021 and 2022 is recognizable in both survey instruments. In addition, the circulation of RSV in Germany – according to ARE surveillance – was longer in the fall of 2021 than in Saxony, and started slightly earlier in the fall of 2022 than in Saxony (Figure 1 (Fig. 1)).

The results of the reporting data from Saxony over the years and the calendar weeks within years are in good accordance with the ARE surveillance data. Both systems show – in agreement with the data from other countries [1], [4] – a higher incidence from 2021 as well as a shift of the main disease phase to early winter after the COVID-19 pandemic. According to the extrapolation of an incidence of approx. 150/100,000 in Saxony to the Federal territory, more than 120,000 RSV reports per year are to be expected. 

## Discussion

### Requirements and options of the Infection Protection Act

The new RSV reporting obligation is a laboratory reporting obligation for RSV detections by name (§ 7 IfSG).

In principle, the Infection Protection Act provides the options of mandatory physician reporting (Section 6 IfSG), mandatory laboratory reporting (Section 7 IfSG) – in each case by name or not by name – and sentinel surveillance (Section 15), with the latter being supplemented in 2022 by the option of wastewater testing [7].

A reporting obligation by name should be limited to reports that “require an immediate response by the public health department to start measures to contain an acute risk of further spread” [17]. A non-named reporting obligation was introduced for pathogens “for which the public health department does not take immediate action in individual cases” and for “data that serve to assess the epidemic situation and derive general preventive measures” [17]. Sentinel examinations were recommended: “if a disease is particularly common, the reporting of each individual case would place an unreasonable burden on the reporting systems and the detection of a disease or infection does not require immediate action by the public health service” [17]. Both of these conditions apply to RSV infections.

Both a non-named laboratory reporting requirement and a sentinel investigation could be suitable for recording data and trends. The total coverage aimed for in mandatory reporting is generally not achieved. As reporting data is collected in relation to the population, incidences per 100,000 can be calculated. Sentinel examinations have a higher specificity compared to mandatory reporting, as a systematic clarification of suspected cases takes place and prevention-relevant information is also systematically recorded. It is possible to calculate the investigation rate (proportion of investigated to suspected cases) and the confirmation rate (proportion of confirmed to suspected cases). Sentinel examinations can therefore not only be used to determine trends as with the (non-named) anonymous mandatory reporting, but also to determine risks and evaluate the success of prevention and vaccinations, as well as to identify new potential hazards at an early stage [17], [18], [19], [20]. 

In view of the frequency of RSV disease, sentinel surveillance is superior to mandatory reporting, partly because sentinel surveillance is better equipped to investigate not only general trends but also risk factors.

### Requirements of the European Commission and recommendations from experts of the European health authority ECDC

Defined communicable diseases and special health risks must be recorded through epidemiological surveillance in the EU Member States. Therefore, the European Commission published an updated list in 2018 [12], which is based on the decision of serious cross-border threats to health [13]; this replaced the previous list from 1999. The current list includes 57 diseases as well as other “specific health risks” such as nosocomial infections and antimicrobial resistance. RSV is not included in this list.

In addition, a group of researchers from the European Center for Disease Control (ECDC) created a decision tree in 2015 which enables a decision to be made for or against mandatory reporting [14]. This takes international health regulations into account as well as possible international consequences of an infectious disease, the incidence and trend of an infectious disease, the resulting strain on the healthcare system, the possibility of contact tracing and the prevention of further diseases, and the identification of risk factors for the development of further prevention strategies.

Aspects of practicability and feasibility are also included, such as the appropriateness (proportionality) of the workload of the public health system, and the unambiguous identification of the infectious disease based on clear clinical, microbiological, or epidemiological criteria. Legal aspects such as the subsidiarity principle (should the reporting obligation be given priority in order to obtain the necessary information?), as well as data and privacy protection are also considered (see Table 2 (Tab. 2)).

To conclude, neither EU legal requirements nor technical, legal and practicability considerations make mandatory reporting of RSV by name necessary and appropriate.

### Options for individual or general preventive measures by the health authorities

The infectiousness of RSV diseases starts days prior to the onset of symptoms and lasts for 3–8 days [20]. Laboratory tests are usually only ordered after the onset of symptoms; thus, considering 1–2 days for the laboratory tests and reporting to the public health department, the latter usually only becomes aware of the infection at the end of the patient's infectiousness – and individual action to protect against further spread is no longer possible in a reasonable manner.

Furthermore, the possible measures would not differ from the generally recommended hygiene measures [20].

In contrast, information on the general prevention of respiratory diseases, such as the promotion of basic hygiene rules, e.g., hand hygiene, coughing and sneezing etiquette, is probably more successful. Health authorities can and should generally and intensively propagate this before the respiratory infection season in winter – regardless of any evidence or reports of pathogens.

For risk populations in infancy and early childhood (see introduction), the AWMF [3] recommends, in addition to the general precautionary measures, that all contact persons carry out careful hand hygiene, refrain from smoking around children, if possible breast-feed infants, and avoid large gatherings of people and visits to day nurseries as far as possible during the infection period.

RSV vaccinations will play a role as preventive measures in the future, regardless of current IfSG reporting data. RSV vaccines are already approved in Germany [21]. A vaccination recommendation based on a risk-benefit assessment by the Standing Committee on Vaccination at the RKI (STIKO) is still pending. The individual risk of disease and the burden of disease are of central importance [22].

To conclude, mandatory reporting of RSV by name is neither suitable, necessary nor useful for preventive measures.

### Experience with the assessment of the reporting data, with particular regard to vaccination as an influencing factor

The evaluation of reporting data is often difficult or even impossible. Since the start of mandatory reporting of MRSA detections in blood and cerebrospinal fluid cultures, for example, considerable differences in the incidences between the individual federal states have been observed, which cannot be evaluated in view of the reporting system’s lack of additional data, e.g., on hygiene measures or the frequency of blood culture sampling in the facilities of the sixteen individual federal states [15].

A vaccination for infants against rotavirus enteritis has been available in Germany since 2006. In 2013, the STIKO issued the vaccination recommendation for infants from 6 months of age and included the vaccination recommendation in the vaccination calendar. From 2006 to 2013, the incidence of RSV reports in 0–4 year olds fell from 1,442/100,000 to 791/100,000; after the introduction of the vaccination recommendation, it decreased further to approx. 300/100,000. Without knowledge of the vaccination prevalence in the individual years and the vaccination status of the infected children, these data cannot be properly evaluated – especially since the incidence of reported norovirus infections also fell by approx. one-third during this time – without available or recommended vaccination. Against this background and in view of various other (unknown?) influencing factors, the RKI has still not been able to provide a valid estimate of the effect of the vaccination recommendation against rotavirus enteritis for infants based on the reported data 10 years after the introduction of the vaccination recommendation. A contrast is provided by a new publication from Italy [23], where rotavirus detections are not notifiable [24] and vaccination was first recommended in Sicily in 2013 and later also in other regions. On the basis of hospital treatment data, those authors [23] showed that among children up to age 35 months, hospital admissions due to rotavirus enteritis fell significantly after the introduction of vaccination, considering the respective vaccination incidence: with a 1% increase in vaccination rate, hospital admissions decreased by 1.25% [23]. In other words, despite the mandatory reporting of rotavirus infections in Germany, no valid analysis of the effectiveness of this vaccination in preventing severe rotavirus infections in infants and young children requiring hospitalization has yet been presented on the basis of the reporting data. This was achieved in another country without mandatory reporting of rotavirus infections on the basis of hospital discharge data combined with vaccination prevalence. 

Due to the lack of valid additional information, mandatory reporting data are generally not suitable for appropriate causal analyses, such as the effects of a vaccination recommendation. Such correlation analyses are best carried out in the context of sentinels [18], [19].

## Conclusion

The following arguments were used to justify the introduction of mandatory reporting of RSV cases by name:

- Improvement of the data basis (to prevent overloading of the healthcare system)
- Implementation of targeted and early investigation and on-site measures to prevent further spread
- Evaluation of the vaccines after the anticipated approval of an RSV vaccination.

Our considerations have shown that mandatory reporting by name is neither required by the EU Commission nor appropriate according to the criteria of an ECDC expert commission. The data which have been available for years from the mandatory reporting of RSV in Saxony suggests an extremely high number of RSV reports, which would lead to a very high and unreasonable burden on the health authorities and also not be associated with the derivation of targeted measures. In contrast, sentinel surveillance provides reliable data. By increasing the size of the sentinels, representativeness can be achieved not only for Germany as a whole, but also regionally. These sentinels can provide good and reliable data to prevent overloading the healthcare system by not- overburdening the health authorities with reporting data.

In addition, initial experience with wastewater sentinel surveillance regarding RSV has shown that this method is suitable for the timely detection of local and regional RSV infection events [25]. However, it is not possible to derive measures on the basis of wastewater surveillance only, as data on the burden of disease and the impact on the healthcare system is lacking. 

As infected persons are already infectious prior to the onset of symptoms and only for approx. 3–8 days in total, laboratory results can only be expected towards the end of infectiousness or even afterwards, so that targeted and early detection or individual measures to prevent further spread are not possible in individual cases. Prevention through general hygiene measures such as hand washing and hygienic coughing and sneezing, as well as vaccinations, appears to make more sense. These can be propagated by the health authorities as general preventive measures without mandatory reporting, as well as by the recommendations of the AWMF [3] regarding additional preventive measures for at-risk populations.

Notification data are not suitable for assessing vaccines; sentinel data are more suitable for this purpose, in addition to specially designed cohort or case-control studies with standardized recording of vaccination status.

Given the above, mandatory reporting of RSV does not seem appropriate. Instead, the existing sentinel surveillance should be further continued and expanded, possibly supplemented by RSV wastewater monitoring as a component of the epidemiological situation assessment.

## Notes

### Competing interests

The authors declare that they have no competing interests.

### Authors’ ORCID

- Ursel Heudorf: 0000-0002-0050-8272
- Anne Marcic: 0009-0008-3660-040X
- Katrin Steul: 0009-0009-2927-8122

## Figures and Tables

**Table 1:**
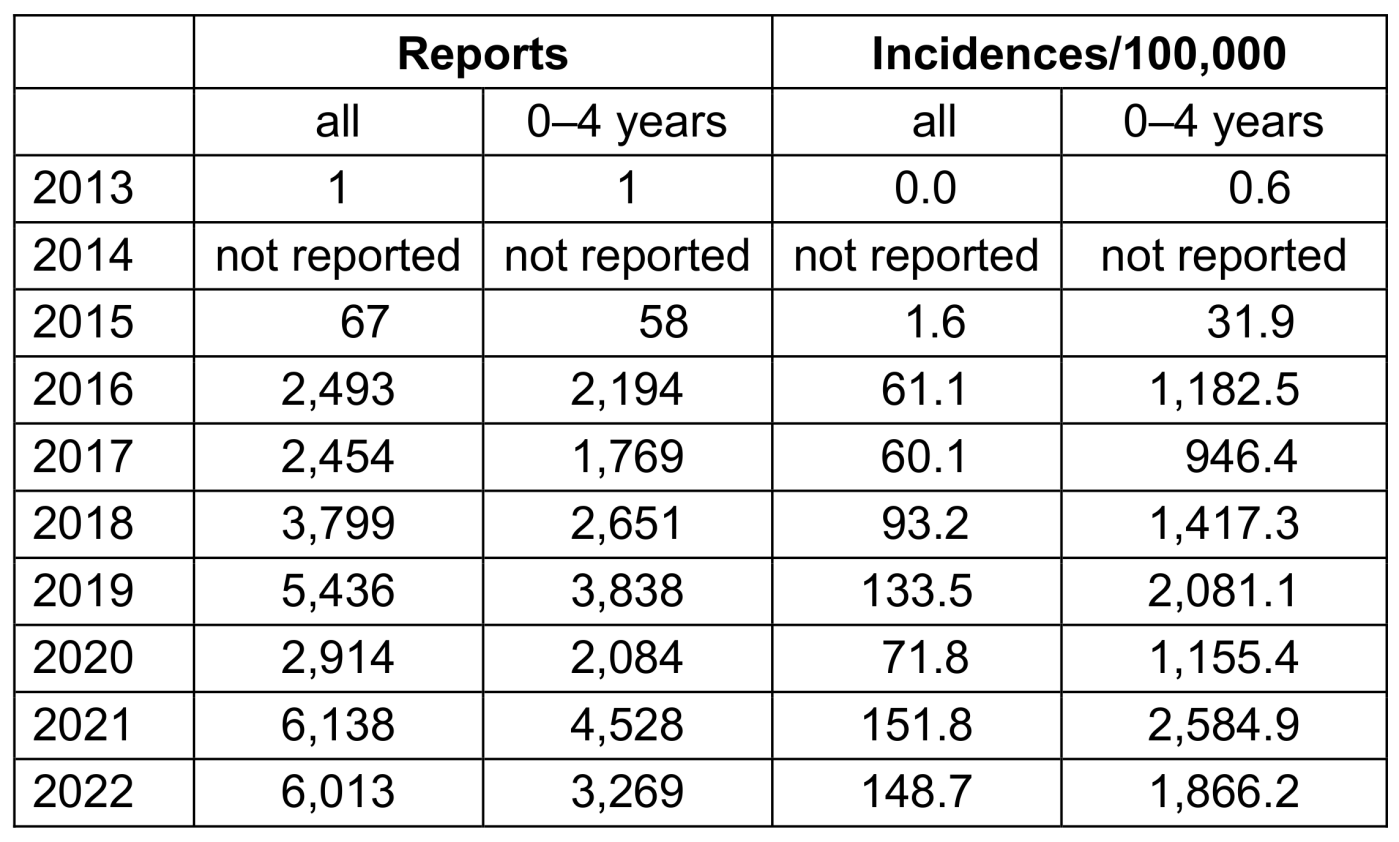
Data on the obligation to report RSV in Saxony [5]

**Table 2:**
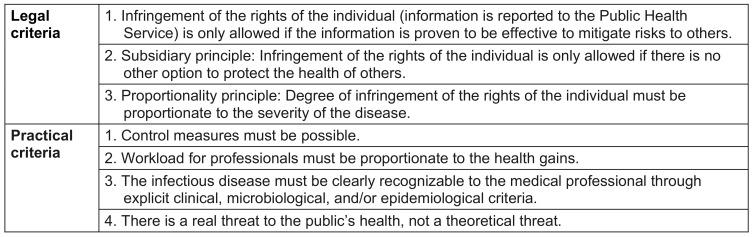
Criteria for mandatory reporting under legal and practicability considerations, considering the contribution to disease control in terms of effectiveness, feasibility or practicability, and necessity (according to [14])
